# Identification of cyanobacterial non-coding RNAs by comparative genome analysis

**DOI:** 10.1186/gb-2005-6-9-r73

**Published:** 2005-08-17

**Authors:** Ilka M Axmann, Philip Kensche, Jörg Vogel, Stefan Kohl, Hanspeter Herzel, Wolfgang R Hess

**Affiliations:** 1 Humboldt-University, Department of Biology/Genetics, Chausseestrasse, D-Berlin, Germany; 2 Humboldt-University, Institute for Theoretical Biology, Invalidenstrasse, Berlin, Germany; 3 Max Planck Institute for Infection Biology, Schumannstrasse, Berlin, Germany; 4 University Freiburg, Institute of Biology II/Experimental Bioinformatics, Schänzlestrasse, Freiburg, Germany

## Abstract

**Background:**

Whole genome sequencing of marine cyanobacteria has revealed an unprecedented degree of genomic variation and streamlining. With a size of 1.66 megabase-pairs, *Prochlorococcus *sp. MED4 has the most compact of these genomes and it is enigmatic how the few identified regulatory proteins efficiently sustain the lifestyle of an ecologically successful marine microorganism. Small non-coding RNAs (ncRNAs) control a plethora of processes in eukaryotes as well as in bacteria; however, systematic searches for ncRNAs are still lacking for most eubacterial phyla outside the enterobacteria.

**Results:**

Based on a computational prediction we show the presence of several ncRNAs (cyanobacterial functional RNA or Yfr) in several different cyanobacteria of the *Prochlorococcus-Synechococcus *lineage. Some ncRNA genes are present only in two or three of the four strains investigated, whereas the RNAs Yfr2 through Yfr5 are structurally highly related and are encoded by a rapidly evolving gene family as their genes exist in different copy numbers and at different sites in the four investigated genomes. One ncRNA, Yfr7, is present in at least seven other cyanobacteria. In addition, control elements for several ribosomal operons were predicted as well as riboswitches for thiamine pyrophosphate and cobalamin.

**Conclusion:**

This is the first genome-wide and systematic screen for ncRNAs in cyanobacteria. Several ncRNAs were both computationally predicted and their presence was biochemically verified. These RNAs may have regulatory functions and each shows a distinct phylogenetic distribution. Our approach can be applied to any group of microorganisms for which more than one total genome sequence is available for comparative analysis.

## Abstract 2

The first genome-wide and systematic screen for non-coding RNAs (ncRNAs) in cyanobacteria. Several ncRNAs were computationally predicted and their presence was biochemically verified. These ncRNAs may have regulatory functions, and each shows a distinct phylogenetic distribution.

## Background

Cyanobacteria constitute a huge and diverse group of photoautotrophic bacteria that perform oxygenic photosynthesis and populate widely diverse environments such as freshwater, the oceans, the surface of rocks, desert soil or the Antarctic. Their existence can be traced back by fossil records possibly up to 3.5 billion years [1].

Because of its small cell size of less than one micron and its requirement for special isolation and cultivation procedures, the marine cyanobacterium *Prochlorococcus marinus *had escaped discovery until just a decade ago [2,3]. In contrast to the majority of cyanobacteria, *Prochlorococcus *shares with *Prochlorothrix hollandica *and *Prochloron *sp. the presence of a protein-chlorophyll *b *complex for photosynthetic light harvesting [4,5]. The presence of chlorophyll *b *had previously been taken as evidence for a separate phylum, the prochlorophyta, to join these three taxa. Molecular evidence has shown, however, that *Prochlorococcus, Prochlorothrix *and *Prochloron *are not closely related to each other [6].

Cyanobacteria of the genera *Prochlorococcus *and *Synechococcus *constitute the most important primary producers within the oceans [7]. Of these, the four marine cyanobacteria, *Prochlorococcus marinus *MED4, MIT 9313, SS120 and *Synechococcus *sp. WH 8102 share a 16S ribosomal RNA identity of more than 97%. In the natural environment, *Prochlorococcus *exists in two distinct 'ecotypes' that thrive at different light optima and constitute distinct phylogenetic clades [8,9]. Thus, the genomes of the low-light-adapted isolates *Prochlorococcus *MIT 9313 and SS120, and of the high-light-adapted MED4 differ by hundreds of genes, facilitating their specialization to different niches within the marine ecosystem [10-12].

An extreme genome minimization occurred in MED4 and SS120 [13], which is thought to be an adaptation to the very oligotrophic and stable environment from which these two strains originated [10,12]. The MED4 strain was isolated from a depth of 5 m in the Mediterranean Sea; its genome of 1.66 megabase pairs (Mbp) encodes 1,716 open reading frames, among them only four histidine kinases, six response regulators and five sigma factors [12]. *Prochlorococcus *SS120 originated from 120 m in the Sargasso Sea [3], and 1,884 predicted protein-coding genes, including five histidine kinases, six response regulators and five sigma factors, have been annotated for its 1.7 Mbp genome [10]. These data indicate a drastically reduced number of systems for signal transduction and environmental stress response (e.g. two-component systems) compared to the larger and more complex genomes of cyanobacteria such as *Synechocystis *sp. PCC 6803 and *Anabaena *sp. PCC 7120, which each harbour 42 and 126 histidine kinases, respectively [14,15]. The small number of regulatory genes in marine *Synechococcus *and *Prochlorococcus *may reflect a more stable environment, in which reactive regulatory responses are less relevant.

It is now becoming increasingly clear that aside from regulatory proteins, bacteria also possess a significant number of regulatory non-coding RNAs (ncRNAs). These are a heterogeneous group of functional RNA molecules normally without a protein-coding function. They are frequently smaller than 200 nucleotides (nt) in size, and act to regulate mRNA translation/decay but can also bind to proteins and thereby modify protein function (for a recent review see [16]). It is well established that such RNAs control plasmid and viral replication [17], transposition of transposable elements [18], bacterial virulence [19], quorum sensing [20] and are important factors in bacterial regulatory networks that respond to environmental changes [21,22]. As a result of recent systematic searches, more than 60 ncRNAs are now known in *Escherichia coli*, most of which had been overlooked by traditional genome analysis [23-28]. Many of these versatile bacterial riboregulators use base pairing interactions to regulate the translation of target mRNAs. Because most of these antisense-acting ncRNAs have only incomplete target complementarity, duplex formation frequently depends on the activity of Hfq, an RNA chaperone, which is structurally and functionally somewhat similar to eukaryotic Sm proteins [29]. Only very recently, an *hfq *homologue was predicted in cyanobacterial genomes, including two of the strains used in this study (*Synechococcus *WH 8102 and *Prochlorococcus *MIT 9313) [29]. This lends support to the idea that riboregulatory processes similar to those of enterobacteria should exist in cyanobacteria.

There is currently no information about the presence of regulatory RNAs and their genes in marine cyanobacteria. Apart from rRNA and tRNA genes, only three other well-characterized RNA genes have been annotated by sequence similarity in each of the four genomes used in this study. These encode the RNA components of RNAse P (M1 RNA), the signal recognition particle (scRNA) and tmRNA (*rnpB*, *ffs *and *ssrA*, respectively). Although the *Prochlorococcus *tmRNA has not been analyzed experimentally so far, it was subject to several *in silico *analyses, predicting it would consist of two separate molecules derived from a common precursor [30,31]. Such a permuted gene structure producing a two-piece mature tmRNA results in a dramatically reduced number of secondary structure elements: only two pairings were predicted in the tRNA-like domain, and a single transient pseudoknot and three other stem-loops were computed for the molecule containing the tag reading frame, whereas the pseudoknot number alone is five in one-piece cyanobacterial tmRNA [30]. It remains unclear, however, what, if any, selective advantage such a simplification in the structural elements of this RNA species would bring. This prompts the question of whether number and complexity of ncRNAs in these organisms is generally reduced as seen with tmRNA and regulatory proteins. And if so, what kind of ncRNAs might have escaped such an elimination and simplification process?

Systematic searches for ncRNAs are still lacking for most eubacterial phyla outside the enterobacteria. Recently, an effective approach to score multiple alignments in terms of secondary structure conservation was suggested [32,33]. Using a comparative genomics approach based on the recently published genome sequences, we have predicted candidates for ncRNAs in four marine cyanobacteria. The expression of these candidate sequences was tested under various growth and stress conditions that are encountered in the natural environment. This resulted in the identification of seven new ncRNAs in MED4, and several homologues in the other three strains.

## Results

### Small RNAs in marine cyanobacteria

**Figure 1:**
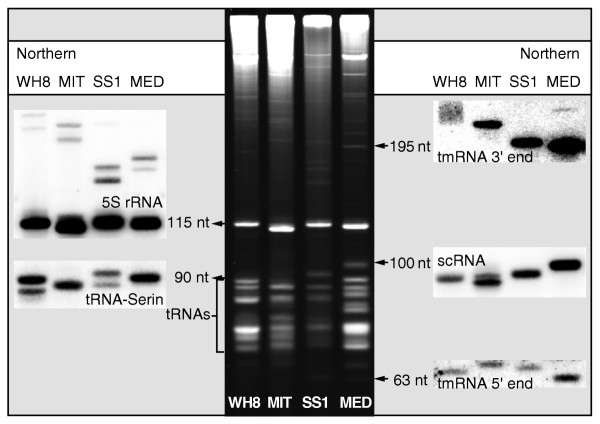
Small RNAs in marine Cyanobacteria. About 10 μg of total RNA from *Prochlorococcus *strains MIT 9313 (MIT), SS120 (SS1) and MED4 (MED) and from *Synechococcus *sp. WH 8102 (WH8) was analyzed by staining a 10% polyacrylamide gel with ethidium bromide (center) and by Northern blot hybridization with DNA-oligonucleotides directed against known RNA molecules such as scRNA (*ffs *gene product), the separate 5' and 3' ends of tmRNA and, as controls, tRNASerin and 5S rRNA. Two distinct precursors of the 5S rRNA were detected. Selected bands have been labeled by arrows in the hybridization and in the gel picture and their sizes (nt, nucleotides) are indicated.

Total RNA samples from the four marine cyanobacteria *Prochlorococcus *MED4, MIT 9313, SS120 and *Synechococcus *WH 8102 were separated on high-resolution polyacrylamide gels to get an overview of the presence of small RNAs. This analysis showed abundant RNA molecules with sizes in the range 50 to 250 nt (Figure 1). A particularly abundant class of RNAs in the 70 to 90 nt size range indicates the location of tRNAs in this gel, which was confirmed by hybridization to the tRNASer [GCU]. The hybridization signal for this tRNA was located at the upper end of this abundant cluster of bands, consistent with the fact that it is the largest annotated tRNA in these genomes. Several small RNAs migrated above the tRNA cluster and very few below it (indicated by the weakly visible bands below the tRNAs). These bands collectively indicated the occurrence of abundant small mRNAs, ncRNAs and precursors to tRNAs and rRNAs.

Eubacterial RNA species, however, very rarely reach a concentration that allows direct identification in a gel. For known RNA species and their possible precursors or degradation products, information on their expression can be gained from hybridization. Here we used oligonucleotide probes for the scRNA and tmRNA and, as controls, the 5S rRNA and tRNASer [GCU], which was predicted to be the tRNA with the highest molecular mass. The lengths of the scRNAs in the four strains vary between 90 and 100 nt, in keeping with the varying lengths of the respective annotated *ffs *genes. The 5S rRNA was detected as a very abundant RNA species together with two precursors. Furthermore, the results of these Northern hybridizations confirmed that *Prochlorococcus *tmRNA is indeed composed of two separate molecules [30].

Several additional bands in the investigated size range indicate the presence of additional abundant small mRNAs or ncRNAs. The lack of specific oligonucleotide probes for hybridisation, however, makes it difficult to get information about these. We thus used a computational prediction to identify candidates for further testing.

### Computational screening and experimental testing identifies novel RNA species

**Figure 2:**
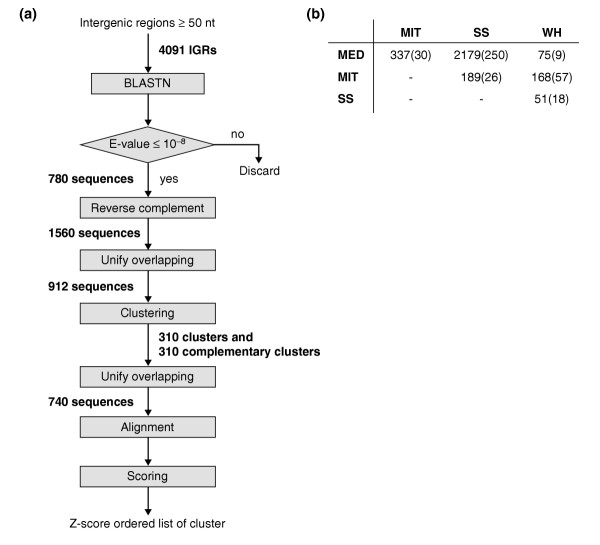
Pipeline for comparative prediction of non-coding RNAs. **(a) **Intergenic sequences (IGRs) longer than 49 base-pairs were gathered from four *Prochlorococcus *and *Synechococcus *genomes and locally aligned using BLASTN. An overview of the intergenic sequences is given in Additional data file 2 (Table S4). Because of the initial asymmetric local alignment using BLASTN (see Figure 2b for a summary of significant BLASTN hits between the strains *Prochlorococcus* MED4 (MED), MIT 9313 (MIT), SS120 (SS) and *Synechococcus* WH 8102 (WH)), all candidate sequences were reverse-complemented. Redundancy in this data set was reduced by unifying those hits from each genome that showed a reciprocal overlap of 85% or greater. This candidate set was used as both query and subject in another local alignment step (BLASTN considering only the query strand as possible subject strand). Sequences that directly produced a significant blast hit (E-value ≤ 10^-10^), or were connected by a chain of such hits, were gathered into clusters ('single-linkage clustering'). Both genome strands were screened; thus, the pipeline produced 310 pairs of clusters in both forward and reverse complementary orientation. After an additional unification step of overlapping sequences within each cluster, the resulting clusters and their complement clusters were scored using ALIFOLDZ [33]. **(b) **The number of BLASTN high-scoring segment pairs for each query and subject combination of intergenic regions is given for a BLASTN E-value cut-off of 10^-5 ^and after import of high-scoring segment pairs with an E-value of 10^-10 ^or lower (in parentheses). MIT, *Prochlorococcus *strain MIT 9313; SS, *Prochlorococcus *strain SS120; WH, *Synechococcus *sp. WH 8102, MED, *Prochlorococcus *strain MED4.

An overview of the computational screening is displayed in Figure 2 and a summary of the highest scoring clusters is given in Table 1. The analysis was basically focused on sequence and structure similarities. Detailed information on all clusters predicted by our method, including the positions of all sequences, is available online [34].

Although the sequence similarities between the predicted RNA elements in cyanobacteria and other organisms were weak, for many of the clusters, clues for their possible function could be obtained from the literature. These included elements that, according to location or structure, might be functionally related to enterobacterial mRNA leader regions mediating the autogenous control of r-protein and rRNA expression (clusters 5, 92, 227, 228) [35,36], the *rpoBC *leader (cluster 245) [37] and the likely terminator (cluster 226). We decided against direct experimental analysis of these elements, which are less likely to be novel types of ncRNAs. Additionally, two possible riboswitches for thiamine pyrophosphate (cluster 2) [38] and cobalamin (cluster 101) [39] were excluded from further experimental investigations.

In the remaining clusters, all candidate sequences from MED4 were tested by Northern hybridization. This restriction was introduced in order to focus the experimental analysis on one particular strain. Each of these seven candidate regions was probed for transcripts from both strands. Three distinct ncRNAs and a group of four related ones yielded strong signals with RNA preparations from MED4. Because some of these ncRNAs have a phylogenetic distribution beyond *Prochlorococcus *(see below), we introduced a more general gene designation, *yfr *(for cyanobacterial functional RNA-coding gene), and Yfr for the respective RNAs. Each of these genes is discussed in detail in the following sections.

### Yfr1: a small RNA encoded between *guaB *and *trxA*

**Figure 3:**
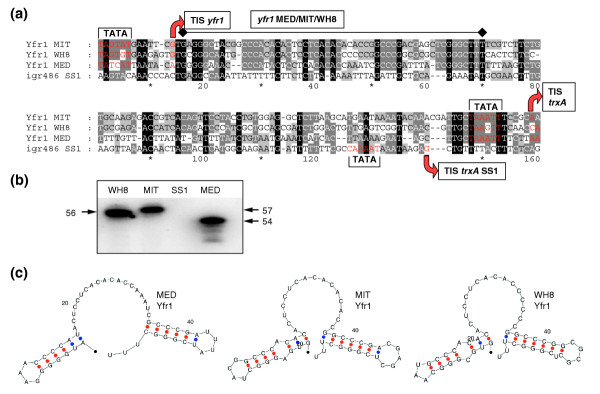
Experimental screen for the presence of an RNA-coding gene in the *guaB-trxA * intergenic region. **(a) **Sequence alignment of the *guaB-trxA *(*guaB*: sequence not shown, located upstream of *yfr1*) intergenic region visualises the conserved *yfr1 *gene labeled by the bar above the alignment and its transcriptional initiation site in three of the analyzed strains (MED, MED4; MIT, *Prochlorococcus *strain MIT 9313; WH8, *Synechococcus *sp. WH 8102) but not in *Prochlorococcus *strain SS120 (SS1). Transcriptional initiation sites (TIS) and the deduced -10 elements are indicated. **(b) **Northern blots show a signal for Yfr1 at a size of 54, 56 and 57 nucleotides (nt) for MED4, WH 8102 and MIT 9313, respectively. No signal with RNA from SS120 confirms the absence of this gene in this strain, as was predicted from the sequence data. **(c) **Predicted secondary structures of Yfr1 in MED4, MIT 9313 and WH 8102 by MFOLD [59].

The *yfr1 *gene was detected in three of the four cyanobacteria in the intergenic region separating *guaB *and *trxA *(Figure 3). In the computational screening only the Yfr1 RNAs from MIT 9313 and WH 8102 were detected with a reasonable Z-score of -3.97 and the MED4 sequence was identified with relaxed BLASTN parameters manually. Although the two adjacent genes *guaB *and *trxA *are located in a similar genomic arrangement in SS120, a *yfr1 *gene was not found at this or any other genomic position nor indicated by a Northern hybridization signal. This result is in agreement with the high sequence divergence of the *guaB*-*trxA *intergenic spacer in SS120 compared to MED4, MIT 9313 and WH 8102.

The direction of *yfr1 *is conserved between MED4, MIT 9313 and WH 8102. It is transcribed in the same direction as the mRNAs from two close-by neighbouring genes, indicating the possibility of cotranscription. Therefore, we searched for the presence of specific transcriptional initiation sites (TIS) for *yfr1 *and for *trxA *by rapid amplification of cDNA ends (RACE). A conserved TIS was mapped for *yfr1*, indicating that this transcript originates from a specific promoter (Figure 3a) and reducing the likelihood that it is cotranscribed with *guaB*. Transcription of the adjacent *trxA *gene, encoding the redox regulator thioredoxin, was found to initiate approximately 100 bp downstream of the 3' end of the *yfr1 *gene (Figure 3a); cotranscription of *yfr1 *with *trxA *is thus unlikely. In SS120, the lack of the *yfr1 *TATA box, and the fact that the *trxA *TIS and TATA box are shifted upstream by about 20 nt compared to the other three strains (Figure 3a), lends additional support for the absence of a *yfr1 *gene.

Compared to other eubacterial ncRNAs [25,40], Yfr1 is one of the shortest bacterial ncRNAs, with a length of only 54, 56 or 57 nt (in strains MED4, MIT 9313 and WH 8102, respectively; Figure 3b). Although direct information on cyanobacterial RNAs is scarce [41,42] and not a single study exists for marine cyanobacteria, the half-lifes of eubacterial mRNAs are frequently in the range of a few minutes. In contrast, Yfr1 is extremely stable as a half-life of more than 60 minutes was measured after transcriptional arrest was induced by rifampicin (see Additional data file 1). No peptide reading frame within *yfr1 *is conserved between any of the three strains, although, as expected for a stable RNA, the three strains that express *yfr1 *share extensive structural conservation. They contain two terminal tetranucleotide loops separated by a 16 to 19 nt unpaired region that contains a CA dinucleotide repeat. Consistently, the 3' located stem-loop element is formed by at least five GC pairs, and is followed by a short stretch of U residues, indicative of a Rho-independent transcription terminator (Figure 3c).

**Figure 4:**
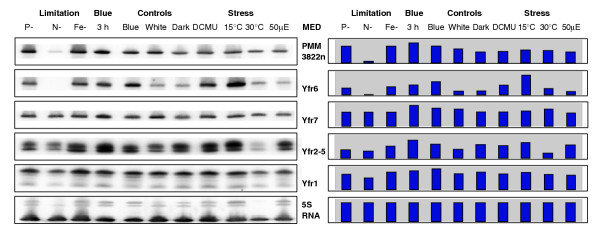
Test of transcript accumulation of Yfr1-7 from MED4 (MED) under different conditions. The left side shows the Northern hybridizations for which the following conditions were used: nutrient depletion (phosphate (P-), nitrogen (N-), iron (Fe-)); blue light for three hours (3 h); controls under blue (Blue), white (White) and no light (Dark); oxidative stress mediated by the application of 3-(3,4-dichlorophenyl)-1,1-N-N'-dimethylurea (DCMU); low (15°C) and high (30°C) temperatures; and high light intensity (50 μE). For comparison, 5S rRNA was hybridized as an internal standard and the mRNA of gene PMM3822n which, with a length of approximately 250 nucleotides, was taken as an example for a small mRNA. Additional controls by quantitative RT-PCR for the genes *isiB *(Fe)*, glnA *(N)*, pstS *(P) and *hli8 *(high light) [data not shown] were carried out to confirm the effects of nutrient depletion or high light. The amounts of these mRNAs were enhanced by a factor of 79.7 (*isiB*), 5.8 (*glnA*), 2.8 (*hli8*) and 4.0 (*pstS*) under the respective treatment compared to standard conditions (data not shown). Yfr6 shows an inconstant signal; for example, at cold, blue/white light, N-, Yfr2 to Yfr5 were hybridized with the consensus oligonucleotide y_gen (Figure 5). The band intensities were quantified and normalized to the amount of 5S rRNA as an internal standard (right).

The expression of many bacterial regulatory RNAs is stimulated by varying environmental cues, and often so by the stress response in which these RNAs then play a role. Therefore, a variety of stress conditions and their possible impact on the accumulation of ncRNAs were tested. Figure 4 shows a series of Northern hybridizations with RNA samples from cells that had been depleted of nitrogen, phosphate or iron, exposed to higher intensities of white or of blue light, or treated with 2 μM 3-(3,4-dichlorophenyl)-1, 1-N-N'-dimethylurea (DCMU) to induce oxidative stress or grown at elevated or lowered temperatures (30°C and 15°C). Normalization of loaded RNA used 5S rRNA as an internal standard to compensate for small RNA sample loading differences; however, Yfr1 levels were unaffected by any of these conditions.

### A new family of related short RNAs

In top scoring cluster 194, a family of structurally highly similar RNAs (Yfr2, Yfr3 and Yfr4) was predicted (Table 1). Subsequent local alignments identified yet another similar sequence in MED4, and at least one homologue each in SS120, MIT 9313 and WH 8102.

**Figure 5:**
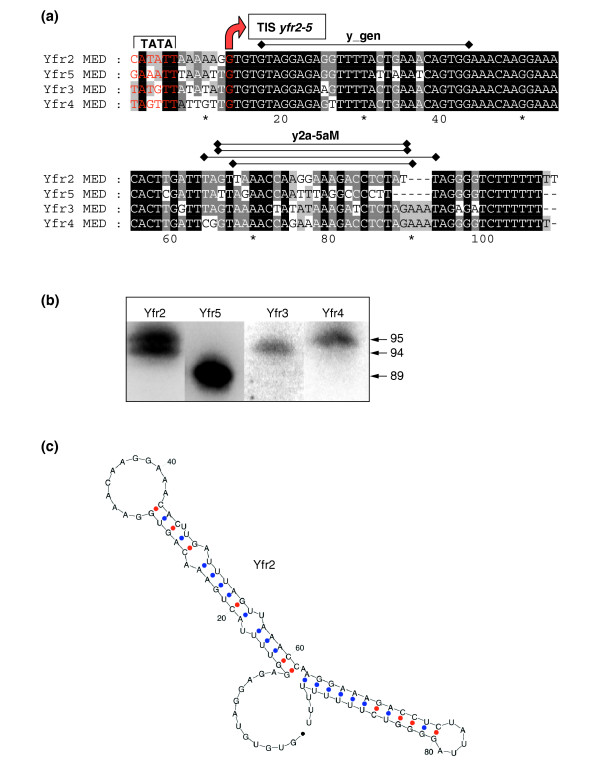
Comparison of Yfr2, Yfr3, Yfr4 and Yfr5 from MED4. **(a) **Sequence comparison of the *yfr2 *through *yfr5 *coding regions of MED4. Transcriptional initiation sites (TIS) and the deduced -10 elements are indicated. The location of specific oligonucleotide probes y2aM, y3aM, y4aM and y5aM used in Figure 5b and in 5' RACE and of the y_gen consensus probe used in Figure 4 is indicated by the lines with black diamonds on the ends on top of the alignment. **(b) **Signals for the four individual non-coding RNAs (ncRNAs) were detected in Northern blots using probes y2aM, y3aM, y4aM and y5aM. These probes have a minimum of five mismatches to their non-target ncRNAs, making cross-hybridizations impossible. The numbers indicate transcript lengths in nucleotides. **(c) **Prediction of secondary structure of MED4 Yfr2 by MFOLD [59].

Northern hybridizations with oligonucleotide probes specific for each of these candidate genes in MED4 yielded distinct bands of 89 to 95 nt. RACE mapping of 5' ends further confirmed that all four loci are transcribed in this organism (Figure 5). The RNAs Yfr2 through Yfr5 in MED4 and their homologues in the other genomes are each encoded by distant genomic loci and the position of their genes is not fixed within the four investigated genomes with respect to adjacent genes (Table 2). The sequence comparison shows that for MED4, Yfr2 and Yfr5 on one hand and Yfr3 and Yfr4 on the other are more similar to each other (Figure 5a). The predicted secondary structures of the Yfr2-5 ncRNA family in MED4 are highly conserved with a GGAAACA repeat within the loop of the predicted 5' hairpin (Figure 5c). Among the different tested environmental conditions, the amount of Yfr2-5 was affected by temperature (up at 15°C and down at 30°C) as well as by nitrogen limitation and incubation in blue light (Figure 4).

### A long RNA in MED4 and SS120

The *yfr6 *gene was predicted in cluster 53 (Table 1). This cluster included nine different sequences (see Additional data file 1, Figure S10), among which only *yfr6 *in MED4 and SS120 may code for a functional RNA. The seven other sequences each have only about 40 nucleotide positions from their respective 5' untranslated region in common with Yfr6. That was sufficient to cluster all nine sequences together, but these other seven sequences included mRNAs for two previously unannotated open reading frames in MED4 and MIT 9313 (PMM3822n and PMT3904n [13]), the three annotated genes Pro0415 (in SS120), SYNW1950 and SYNW2450 (in WH 8102) as well as two more possible open reading frames in WH 8102, (27_W1i1019 and 6_W1i283), which possibly code for peptides with similarity to the first five gene products (see also Figure S10B in Additional data file 1). In contrast, Yfr6 from the two strains each have an extended sequence and structural similarity to each other.

**Figure 6:**
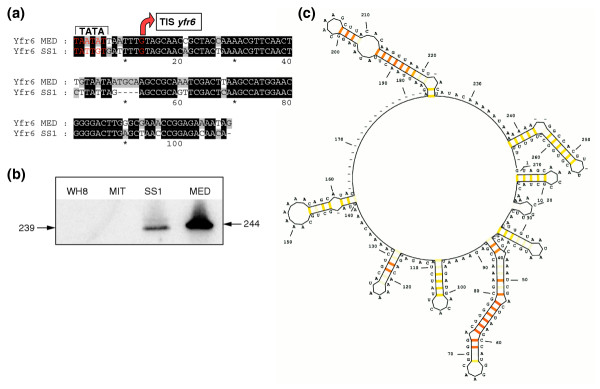
Characterization of a gene encoding Yfr6. **(a) **Sequence alignment of the region containing the transcriptional initiation site (TIS) and the first 97 transcribed nucleotides of Yfr6 from MED4 and SS120. The alignment begins with the TATA element (in red) preceding the mapped first transcribed guanidine (labelled by an arrow). **(b) **In Northern blots, a signal for the predicted Yfr6 was detected for 244 and 239 nucleotides in total RNA from MED4 (MED) and SS120 (SS1), respectively, but not in RNA from WH 8102 (WH8) and MIT 9313 (MIT). **(c) **Comparison of Yfr6 secondary structures using ConStruct version 3.0a [60] The base pairing probability is colour-coded from light yellow (low) to red (high). Missing positions are indicated by a dash. The predicted RNA structures were obtained by RNAfold at 24°C. Both sequences were equally weighted (1.0). The consensus was calculated based on the predicted optimum structures. Default parameters were used for all other options.

In MED4, *yfr6 *is located between the hypothetical PMM0660 gene and PMM0659, the latter encoding 322 amino terminal residues of a DNA ligase. The region is framed by *trnS *and *nrdJ *(encoding a B12-dependent ribonucleotide reductase). In SS120, the *nrdJ-trnS *region lacks the *yfr6 *gene, which instead is located 448 nt downstream of another ncRNA gene, *yfr7*. Despite the different genomic locations, Yfr6 sequences from the two strains show a nucleotide identity of approximately 70% to each other (Figure 6a; Additional data file 1, Figure S10). A Northern blot signal for Yfr6 is restricted to MED4 and SS120 and no signal was found in WH 8102 and MIT 9313 (Figure 6b). This 244 nt RNA had a half-life of approximately 2 minutes in MED4. In MED4, blue light and incubation in the cold elevated the expression of Yfr6 compared to white light or darkness. In addition, expression was reduced upon nitrogen depletion and under high light conditions (Figure 4). The *yfr6 *locus could also code for a 33 amino acid peptide as there is a possible reading frame that is conserved between MED4 and SS120 that begins at nucleotide 97 of the Yfr6 transcript in MED4. This situation, a relatively long transcript with strong structural potential (Figure 6c) and a very short centrally located reading frame, resembles the RNAIII from *Staphylococcus aureus*, a riboregulator from which the 26 amino acid δ-hemolysin peptide is also translated [43]. In the hyperthermophilic archaeon *Sulfolobus solfataricus*, recently as many as 13 sense strand RNA sequences have been found that were encoded either within, or overlapping, annotated open reading frames [44].

### Yfr7 exists in 11 different marine cyanobacteria

**Figure 7:**
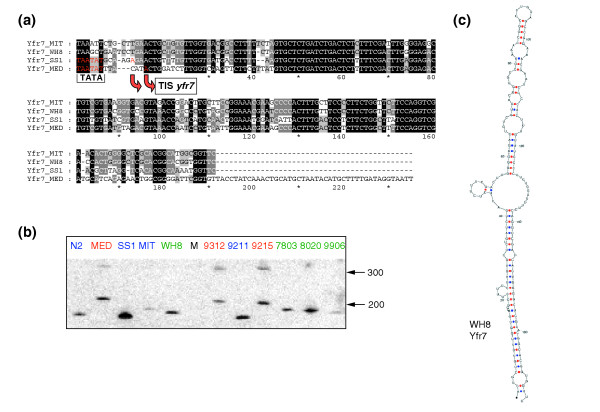
Characterization of Yfr7. **(a) **Sequence alignment of Yfr7 from four marine cyanobacteria. The 5' end transcriptional initiation site (TIS) was mapped for Yfr7 from MED4 and SS120. **(b) **In Northern blots, a signal for the predicted Yfr7 was detected with RNA from all four strains and seven additional strains from the marine cyanobacterial radiation: *Prochlorococcus *NATL2A (N2), MED4 (MED), SS120 (SS1), MIT 9313 (MIT), MIT 9312 (9312), MIT 9211 (9211), MIT 9215 (9215) and *Synechococcus *WH 8102 (WH8), WH 7803 (7803), WH 8020 (8020), RS9906 (9906). The high-light-adapted *Prochlorococcus *strains are labelled in red, low-light-adapted strains in blue, and *Synechococcus *strains are colour-coded in green; M indicates the marker lane. Numbers indicate lengths of RNA markers in nucleotides. **(c) **Prediction of secondary structure of the *Synechococcus *WH 8102 ncRNA Yfr7.

The *yfr7 *gene is located downstream of *purK *(encoding phosphoribosylaminoimidazole carboxylase) in all four strains analyzed here (Table 2). At first, our search strategy identified this gene only in MED4 and SS120 (Table 1), due to the fact that in MIT 9313 and WH 8102 this corresponding region is located within annotated mRNA genes. These hypothetical genes, PMT0670 in MIT 9313 and SYNW1307 in WH 8102, are annotated on the forward strand. We did not detect their expression, but found strong signals for Yfr7, which is transcribed from the complementary strand. The sequence of Yfr7 is highly conserved between the four strains (Figure 7a). Rifampicin tests showed this RNA to be stable (half-life >1 h). In MED4, expression of Yfr7 was not affected by conditions employed in Figure 4.

Its high sequence conservation enabled us also to define oligonucleotides that hybridized to this RNA in four additional, unsequenced strains of *Prochlorococcus *and in three additional *Synechococcus *strains (Figure 7b). The signal pattern is very distinct as all three *Prochlorococcus *strains adapted to high light (MED4, MIT 9312, MIT 9215) have two signals in hybridization, one at approximately 200 nt and one at approximately 300 nt, whereas RNA from the four low-light-adapted *Prochlorococcus *(SS120, MIT 9313, NATL2A and MIT 9211) and four *Synechococcus *(WH 8102, WH 7803, WH 8020, RS9906) strains gave a single signal at approximately 175 to 185 nt (Figure 7b). These strains represent a large genetic diversity within the marine cyanobacterial radiation [45], thus the presence of orthologues of *yfr7 *in additional and even more distant cyanobacteria appeared likely. Indeed, in the freshwater cyanobacteria *Synechococcus *PCC 6301 and *Synechocystis *PCC 6803, a 6Sa (or SsaA) RNA has also been described, which is located directly downstream of *purK *[46]. There is some structural similarity between Yfr7 and the 6Sa RNA, which leads us to assume that these RNAs are homologues of each other. In addition, a recent publication provided comparative structural information suggesting that the ncRNA Yfr7 we describe here and SsaA or 6Sa RNA from the latter cyanobacteria have structural elements in common with the 6S RNA of γ-proteobacteria, in particular a large internal loop (the central bubble in Figure 7c), a typical closing stem and terminal loop [47]. This possibly indicates that the here described Yfr7s are the orthologues of γ-proteobacterial 6S RNA and may have a similar role throughout the whole eubacterial radiation.

## Discussion

The genomes of *Prochlorococcus marinus *SS120, MIT 9313, MED4 and *Synechococcus *WH 8102 provide a unique dataset for cyanobacterial genome analysis. These genomes differ by several hundred genes from each other, yet most of the operons and gene clusters present in more than a single genome are co-linear [10-12]. Furthermore, the *Synechococcus/Prochlorococcus *group is very well investigated with regard to their global significance in the marine ecosystem, and there is clear evidence for speciation processes in terms of specific ecological niches, the position in phylogenetic trees, and the presence of more or less derived features (for a review, see [7]). Although there is no well established genetic system for *Prochlorococcus *to test gene functions directly, these features collectively make these cyanobacteria emerging model organisms for marine photoautotroph bacteria.

In certain other eubacteria such as *E. coli *and *Vibrio cholerae*, several ncRNAs were demonstrated to be essential regulatory factors mediating rapid responses to environmental changes. The underlying regulatory mechanisms range from antisense binding to mRNAs to direct sensing of metabolites, as it is the case with riboswitches. For free-living marine phototrophs such as the cyanobacteria investigated here, regulatory circuits involving ncRNAs can be expected too. However, except for RNase P RNA, scRNA and tmRNA, the three ncRNAs that are easiest to identify, little had been known about ncRNAs genes in these marine cyanobacteria. In a broader context, information has remained scarce on riboregulators and RNA-coding genes even for the group of cyanobacteria as a whole.

Using an elaborate biochemical protocol, a single ncRNA was previously identified in the freshwater cyanobacteria *Synechococcus *PCC 6301 and *Synechocystis *PCC 6803 [46]. In addition, mapping of transcriptional units within the gas vesicle operon of *Calothrix *identified a single antisense transcript [48]. Here, we report the presence of new non-coding RNAs in the group of marine unicellular cyanobacteria with a focus on *Prochlorococcus marinus *MED4. Several more ncRNA candidate genes were predicted in the two relatively larger genomes of WH 8102 and MIT 9313 but still await experimental testing. An overview of the candidate regions identified by our screen is presented in Table 1 and a summary of the experimentally confirmed new ncRNAs is presented in Table 2. In addition to the identification of ncRNAs, the computational results indicated the presence of conserved secondary structure elements relating to the upstream untranslated regions of several r-protein operons. Thus, autogenous control mechanisms over the expression of these operons, similar to those in enterobacteria [35,36] may exist in these cyanobacteria.

The percentage of true RNA elements and ncRNAs found in our screen is very high, whereas the number of predicted ncRNA genes above the Z-score cut-off was low in MED4. It is likely that additional candidate ncRNAs have escaped detection. The performance of the computational algorithm is sensitive to the number of sequences. One important limitation in this context relates to the focus on RNA structures that are additionally conserved by primary sequence. Furthermore, because of the restriction to intergenic regions, ncRNAs that reside within annotated regions will be missed. This affects the whole class of antisense RNAs that are encoded complementary to their target. Also, misannotations may reduce the number of sequences in a cluster, like in the case of *yfr7*, which is in a region in which a reading frame was annotated on the complementary strand in two of the genomes investigated here. Indeed, in a test using an alignment of Yfr7 from all four species, an improved Z-score of -7.8 was detected.

**Figure 8:**
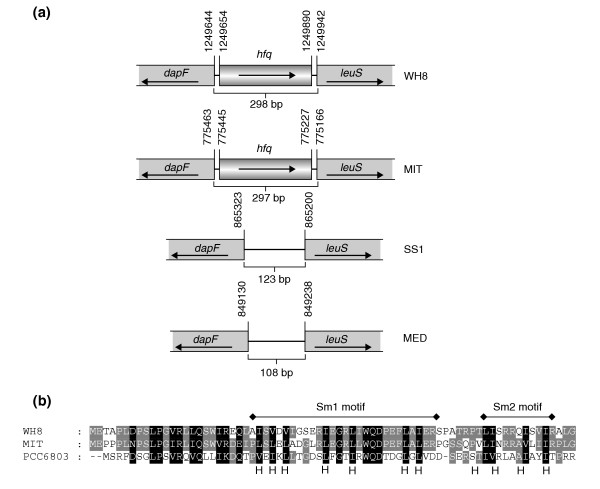
A putative gene encoding the RNA chaperone Hfq can be predicted in two of the four marine cyanobacteria investigated here. **(a) **The *dapF-leuS *intergenic region in *Synechococcus *WH 8102 (WH8) and *Prochlorococcus *MIT 9313 (MIT) is, at 298 and 297 nucleotides, respectively, relatively long and contains a short reading frame for a putative *hfq *gene. In *Prochlorococcus *SS120 (SS1) and MED4 (MED), this region is only 123 and 108 nucleotides, respectively. **(b) **Sequence comparison of putative Hfq proteins from the three cyanobacteria *Synechocystis *PCC 6803 (ssr3341 gene), *Synechococcus *WH 8102 (WH8) and *Prochlorococcus *MIT 9313 (MIT). Hydrophobic residues within the Sm1 and Sm2 motifs [29] are indicated by an H.

Our analysis did reveal an interesting set of structural elements. Especially for MED4 and SS120, which underwent a strong genome reduction, the ncRNAs found may be of considerable importance. Both WH 8102 and MIT 9313 contain a *hfq *gene, whose product has been shown to be intimately linked to the activity of small regulatory RNAs in enterobacteria [29]. Intriguingly, there is no *hfq *gene in SS120 or MED4, although the genomic region flanking *hfq *is otherwise conserved among the four species (Figure 8). It is likely that, together with *hfq*, several ncRNA genes have been deleted during the evolution of the *Prochlorococcus *group towards the minimal genome. Thus, those ncRNAs still remaining in an organism such as MED4 must have been subject to strong positive selection and may act independently of Hfq. (It is worthwhile noting that in *E. coli*, only 30% of investigated ncRNAs have been shown to be bound by Hfq [28].)

The functions of these ncRNAs are currently unclear. The mode of action of ncRNAs supposed to act through an antisense mechanism can be studied by transferring the ncRNA as well as the putative target(s) to an appropriate host or model organism. For unicellular marine cyanobacteria, *Synechococcus *WH 7803 might become such a model as its genome analysis has almost completely been finished [49] and because there is a genetic system. Those ncRNAs with orthologues over a wider phylogenetic distance could be functionally analyzed also directly in cyanobacteria for which well-established genetic tools exist, such as *Synechococcus *PCC 7942 or *Synechocystis *PCC 6803. A very good candidate is Yfr7, which is likely to be present in all cyanobacteria and could be the orthologue of γ-proteobacterial 6S RNA [47]. 6S RNA is required for the repression of σ^70^-dependent promoters under nutrient limitation and concomitant activation of certain σ^S^-dependent promoters [50]. Cyanobacteria do not harbor an obvious orthologue of the enterobacterial stationary phase sigma factor σ^S^. Therefore, it remains to be shown if Yfr7/SsaA/6Sa RNA is also functionally related to γ-proteobacterial 6S RNA. But the widespread occurrence of this ncRNA opens exciting opportunities to test the function of Yfr7 directly in cyanobacteria.

Evidence of function may further come from the comparison of expression patterns, structures as well as genomic location, and from the presence or absence of a given ncRNA gene in the different strains. For instance, *yfr1 *might be dispensable for growth at greater depths; this gene is clearly absent from the ultra low-light-adapted SS120 but is present in the other three cyanobacteria, whereas Yfr2 through Yfr5 are in length and the degree of mutual identity similar to four ncRNAs implicated in quorum sensing in *Vibrio *species [20]. Consequently, the ncRNAs identified here may constitute important regulatory or structural components of a free-living marine cyanobacterium.

## Conclusion

The first genome-wide and systematical screen for ncRNAs in cyanobacteria is provided. Genes encoding functional RNAs are notoriously difficult to predict during standard annotation of microbial genomes. Here, we took a comparative computational approach that was based on sequence and structure conservation as was recently introduced for the identification of eukaryotic ncRNAs [33]. In view of the rapidly growing number of microbial genome sequences, such screens that are based on comparative analysis will become increasingly possible. We have analyzed the highest scoring candidates of the prediction further and detected several previously unknown ncRNAs as well as other elements that function at the RNA level. The list of high scoring candidates contained a very low rate of true negatives. This indicates two points: first, the employed method is very efficient in finding microbial ncRNAs and other RNA elements. Although we already used a soft cut-off value, however, an even lower limit might be used for microbial genomes such as those analyzed here. Second, the 17 ncRNAs detected here in MED4, SS120, MIT 9313 and WH8102 are only a part of the total ncRNA population present in these species. Thus, our data indicate that it is very likely that ncRNAs play an important regulatory and structural role in cyanobacteria. Consequently, they deserve more attention in view of the important function these microbes play in the global ecosystem.

## Materials and methods

### Cultivation of cyanobacteria

Cultures of *Prochlorococcus *and *Synechococcus *were grown in artificial sea water medium (*Prochlorococcus *MED4, NATL2A-MIT and *Synechococcus *WH 7803, RS9906, WH 8102) [51], or based on Atlantic seawater in PRO99 media (SS120, MIT 9313, MIT 9312, MIT 9211, MIT 9215) [52] under 18 (MED4, MIT 9312, MIT 9215, WH 7803, RS9906 and WH 8102) or 10 (all other strains) μmol quanta m^-2^s^-1 ^white light at 23°C in a 12 h day-12 h night cycle.

*Prochlorococcus *MED4 was subjected to various environmental perturbations by depletion of nitrate, phosphate, iron in artificial seawater; a shift from approximately 10 μmol quanta m^-2^s^-1 ^white light into darkness or into 10 μmol quanta m^-2^s^-1 ^blue light or into 50 μmol quanta m^-2^s^-1 ^daylight as high light condition, or the addition of DCMU to a final concentration of 2 μM for the inhibition of photosynthetic electron transport and the induction of severe oxidative stress; as well as temperature shifts to 15°C or 30°C. MED4 cultures were concentrated ten-fold by centrifugation for 10 minutes at 9,000 rpm at 15°C to 20°C and cell pellets were washed once with the corresponding depleted media if necessary. The concentrated cultures were incubated for 3 h at the respective condition.

### RNA analysis

Total RNA was isolated as previously described [53] but with modified lysis conditions for MIT 9313 and WH 8102 as these strains gave poor RNA yields using the standard procedure. The resuspended cells from these strains were homogenized in Z6 buffer [54] by several freeze-thaw cycles using liquid nitrogen over a time of 30 minutes, followed by the addition of one volume of acidic phenol and incubation at 60°C for another 30 minutes. Total RNA was separated in 10% polyacrylamide-urea gels. Polyacrylamide gels were stained with ethidium bromide (0.3 μg/l) in 1 × TBE buffer [55], rinsed with water and analyzed with a Lumi-Imager F1 system (Roche, Mannheim, Germany). Transcript sizes were determined by correlation to *Msp*I-digested DNA of plasmid puc19. Mapping of RNA 5' ends was performed by rapid amplification of cDNA ends as described [24]. We verified in three different ways that the same amounts of RNA samples were loaded in Northern blots: first, by measurement of RNA concentrations; second, by direct comparison of rRNA band intensities after staining by ethidium bromide; and third, by control hybridizations using the 5S rRNA as an internal standard.

To determine RNA stability, cells were treated with rifampicin (200 μg/ml; SIGMA, Munich, Germany) and filtered rapidly (within 60 s) through Supor 0.45 μm membrane filters (PALL, Dreieich, Germany) at different time points after treatment, transferred in resuspension buffer (10 mM NaOAc, pH 4.5, 200 mM sucrose, 5 mM EDTA) and frozen in liquid nitrogen. RNA was isolated by dissolving the filter in acidic phenol at 60°C followed by standard phenol-chloroform extraction as described above. Gel-separated RNAs were electroblotted to Hybond-N+ membranes (Amersham, Freiburg, Germany). Following prehybridization for at least 10 minutes in 50% deionized formamide, 7% SDS, 250 mM NaCl and 120 mM Na(PO_4_) pH 7.2 at 45°C, oligonucleotide probes labelled by polynucleotide kinase with 30 μCi γ^32^P-ATP were added and hybridized at 52°C for at least 4 h (except for the probes designed for Yfr2 through Yfr5, which were hybridized at 45°C and washed at 40°C). All DNA oligonucleotides are listed in the Additional data Table S3. The membranes were washed in 2 × SSC (3 M NaCl, 0.3 M sodium citrate, pH 7.0) [55], 1% SDS at 45°C for 10 minutes; 1 × SSC, 0.5% SDS at 45°C for 5 min; and briefly in 0.1 × SSC, 0.1% SDS at ambient temperature. Signals were detected and analyzed on a Personal Molecular Imager FX system with Quantity One software (BIO-RAD, Munich, Germany).

### Computational methods

To identify candidates for our experimental investigations, we took a comparative computational approach that was based on sequence and structure conservation and used the program ALIFOLDZ [33]. The genome sequences of *Prochlorococcus *SS120, MED4, MIT 9313 and *Synechococcus *WH 8102 were used in the versions given in Additional data file 2 (Table S4). A summary of the computational screening is given in Figure 2 and a complete list of parameters is available in Additional data file 2 (Table S5).

We assumed that homologous RNA structures would show a reasonable degree of conservation on the sequence level for the given set of genomes. BLASTN (Version 2.2.8 [56]) was used to screen for local sequence conservations within intergenic spacer regions (IGRs) longer than 49 bp. These were defined as those regions not overlapping any annotated CDS, rRNA, tRNA or misc_RNA feature (primary tags according to EMBL feature table definition [57]) on either strand. An overview of some characteristics of the intergenic sequences is given in Additional data file 2 (Table S4). Because sequence conservation concerns both DNA strands and because the local alignment was done asymmetrically (e.g. MED4 IGRs were aligned versus MIT 9313 IGRs, but not vice versa; Figure 2B), all hit sequences were reverse complemented.

ALIFOLDZ shows increased sensitivity with the number of aligned sequences [33]. Thus, to take advantage of a multi-genome comparison, we transformed the pairwise sequence alignments into multi-sequence clusters via single-linkage clustering. Before proceeding to single-linkage clustering, redundancy was reduced by unifying those hits from each genome that showed a maximum reciprocal overlap of 85% or greater. The reduced sequence set was used as both query and subject set in another local alignment step (BLASTN considering only the query strand as possible subject strand). Sequences that produced a significant blast hit (E-value ≤ 10^-10^) for a given query were collected into initial clusters. These were unified if they contained at least one common sequence. The procedure produced a total of 310 clusters plus 310 clusters with the reverse complement of these sequences. Candidate sequences that overlap less than the previous coverage cut-off of 85% but are long enough to produce significant BLASTN hits can result in duplicate sequences within clusters. These may negatively affect the alignment and scoring. Therefore, these sequences were merged within each individual cluster using a less restrictive reciprocal coverage cut-off (≥10%).

Finally, each cluster was aligned using CLUSTALW (Version 1.81, default parameters) [58] and the resulting alignments were scored by ALIFOLDZ. Also, single sequence clusters were scored by ALIFOLDZ (by normalized minimum fold energy). As the scoring method is, besides any biological limitations, sensitive to the number of sequences in the alignment, we considered the Z-score cut-off of -4 used by Washietl and Hofacker [33] as a soft cut-off for both alignments and single sequences. For all structure computations, folding temperatures were set to 24°C, which is the approximate habitat temperature of the marine cyanobacteria studied here [7].

Despite any structural conservation, any RNA in principle may encode for a peptide. The necessary reading frame as defined in this analysis consisted of at least ten consecutive codons starting with either of the possible start codons ATG, GTG, TTG or ATT and finishing with TAA, TAG or TGA. If a reading frame was present, the possible conservation of the encoded peptide sequence amongst other cyanobacteria was evaluated by alignments. Only in the case of a conserved open reading frame did we consider the RNA to be coding.

If not indicated otherwise, all individual secondary structure predictions were done using MFOLD [59].

## Additional data files

The following additional data are available with the online version of this article. Additional data file 1 includes figures showing the determination of half-lifes for several ncRNAs (Figure S9) and the composition of cluster 53 (Figure S10). Additional data file 2 includes tables listing the genome versions used in this study and details of intergenic regions (Table S4), the parameters for the initial local alignment of intergenic spacer regions, the clustering step and ALIFOLDZ (Table S5) and the sequences of oligonucleotides used in this study (Table S3). Furthermore, detailed information on all clusters predicted by our method including the positions of all sequences is available online [34].

## Supplementary Material

Additional data file 1 Figures showing the determination of half-lifes for several ncRNAs (Figure S9) and the composition of cluster 53 (Figure S10). Click here for file

Additional data file 2 Tables listing the genome versions used in this study and details of intergenic regions (Table S4), the parameters for the initial local alignment of intergenic spacer regions, the clustering step and ALIFOLDZ (Table S5) and the sequences of oligonucleotides used in this study (Table S3). Click here for file

## Figures and Tables

**Table 1:** List of high scoring clusters

CLID	Sequence number	Strain				Alignment length	Z	Z rev	Exp	Comment	Reference
		MED	SS1	MIT	WH8						

194	3	3	-	-	-	201	-6.28	**-12.94**	+	*yfr2*, *yfr3*, *yfr4*	This paper
5	3	-	1	1	1	345	-7.58	**-10.18**	NT	*rplCD *operon leader, corresponds to *Escherichia coli *S10 r-operon	[61, 62]
92	5	1	1	1	2	756	-4.47	**-9.9**	NT	*rrn *operon leader	[63, 64]
112	2	2	-	-	-	1129	-8.15	**-9.15**	NT	Reciprocal coverage of 7.9%, artifact due to low-complexity sequences	-
229	2	-	-	1	1	161	**-7.98**	-4.90	NT	Hghly similar sequences, putative ncRNAs	This paper
227	2	-	-	1	1	229	**-7.38**	-7.32	NT	*rplJ *operon leader, corresponds to *E. coli *β r-operon	[65]
84	1	-	1	-	-	122	**-6.27**	-5.54	+	*Yfr2*	This paper
101	3	-	1	1	1	152	**-5.77**	-5.61	NT	Putative Cobalamin riboswitch	[39, 66]
226	2	-	-	1	1	142	**-5.29**	-5.28	NT	Possible bi-directional terminator of the *rplKAJL *operon	Predicted by TransTerm [67]
9	6	-	1	1	4	397	-4.38	**-4.95**	NT	No conserved position, no significant BLASTN hit to MED4	This paper
51	2	1	1	-	-	146	-0.84	**-4.92**	*+*	*yfr7*	This paper
53	9	2	2	1	4	697	-3.26	**-4.59**	+	*yfr6 *in MED4 and SS120 and a subgroup of 5' UTR regions to annotated genes and putative unannotated genes in all four strains	This paper
245	2	-	-	1	1	259	-3.7	**-4.53**	-	*rpoBC *operon leader, corresponds to *E. coli *attenuator separating the *rp*l genes from *rpoBC *in the *rplKAJLrpoBC *gene cluster	[37, 68]
217	1	1	-	-	-	153	-1.63	**-4.28**	-	Located between genes for a two-component sensor histidine kinase and a conserved hypothetical protein	This paper
87	2	-	-	1	1	336	**-4.24**	-3.64	NT	Region upstream of the *rbcLS *cluster containing conserved promoter	[51]
228	2	-	-	1	1	106	-0.67	**-4.00**	NT	*Rpl11 *operon leader, corresponds to *E. coli *L11 r-operon	[69, 70]
257	2	-	-	1	1	176	-3.42	**-3.97**	+	*Yfr1*	This paper
2	3	-	1	1	1	197	**-3.93**	-2.94	NT	Putative TPP riboswitch in front of *thiC*	[38]

RNA elements were predicted according to the scheme shown in Figure 2. The total number of sequences in each cluster and the distribution within the four compared genomes plus the total alignment length are given. The elements are ordered according to the lowest score in either forward (Z) or reverse (Z rev) orientation (in bold letters). The lower the Z-score the higher the support for structural conservation. Exp (experimental testing): +, tested positively by Northern hybridisation; NT, not tested. The cluster identities (CLID) were also used in Table 2. For further details and exact positions of sequences see Table 2 and [34].

**Table 2:** Summary of identified ncRNA genes in *Prochlorococcus *MED4 and their orthologues in three related strains of marine cyanobacteria

Strain	RNA gene name	CLID	Coordinates of RNA gene	Length of RNA in nucleotides	Adjacent protein-coding genes	Orientation
**MED4**	yfr1	257	Complement (1000744..1000797)	54	*trxA *and *guaB*	← ← ←
	yfr2	194	346828..346921	94	*PMM0363 *and *PMM0364*	→ → →
	yfr3	194	654511..654604	95	*PMM0686 *and *PMM0687*	→ → ←
	yfr4	194	383389..383483	94	*PMM0404 *and *phdC*	← → →
	yfr5	NP	Complement (972088..972176)	89	*PMM1027 *and *PMM1028*	→ ← →
	yfr6	53	Complement (627729..627972	244	*PMM0659 *and *PMM0660*	→ ← ←
	yfr7	51	652625..652844	220	*purK *and *PMM0684*	→ **→ → **
**SS120**	yfr2	84	Complement (556612..556701)	90	*rpsU *and *Pro0591*	← ← ←
	yfr6	53	Complement (923780..924018)	239	*Pro1007 *and *purK*	→ ← ←
	yfr7	51	Complement (924466..924640)	175	*Pro1007 *and *purK*	→ ← ←
**MIT 9313**	yfr1	257	1220973..1221029	57	*guaB *and *trxA*	→ → →
	yfr2	NP	Complement (1667304..1667390)	87	*PMT1567 and PMT1568*	→ ← ←
	yfr7	NP	727045..727219 (complementary to *PMT0670*)	175	*purK *and *PMT0671*	→ → ←
**WH 8102**	yfr1	257	Complement (706826..706881)	56	*trxA *and *guaB*	← ← ←
	yfr2	NP	1127972..1128056	NT	Overlapping *SYNW1139*	
	yfr3	NP	1131773..1131856	NT	*SYNW1140 *and *SYNW1141*	→ → ←
	yfr7	NP	Complement (1302885..1303058) (complementary to *SYNW1307*)	174	*SYNW1306 *and *purK*	→ ← ←

The genome positions and names of protein coding genes refer to the genome versions indicated in the Additional data file 2 (Table S4). The cluster identifier (CLID) is identical to that used in Table 1. NP, not directly predicted by the pipeline; NT, not experimentally tested.

## References

[B1] Schopf JW (1993). Microfossils of the early Archean Apex chert: new evidence of the antiquity of life.. Science.

[B2] Chisholm SW, Olson RJ, Zettler ER, Waterbury JB, Goericke R, Welschmeyer N (1988). A novel free-living prochlorophyte abundant in the oceanic euphotic zone.. Nature.

[B3] Chisholm SW, Frankel SL, Goericke R, Olson RJ, Palenik B, Waterbury JB, West-Johnsrud L, Zettler ER (1992). *Prochlorococcus marinus *nov. gen. nov. sp.: an oxyphototrophic marine prokaryote containing divinyl chlorophyll *a *and *b*.. Arch Microbiol.

[B4] LaRoche J, van der Staay GW, Partensky F, Ducret A, Aebersold R, Li R, Golden SS, Hiller RG, Wrench PM, Larkum AW, Green BR (1996). Independent evolution of the prochlorophyte and green plant chlorophyll *a/b *light-harvesting proteins.. Proc Natl Acad Sci USA.

[B5] Chen M, Hiller RG, Howe CJ, Larkum AW (2005). Unique origin and lateral transfer of prokaryotic chlorophyll-b and chlorophyll-d light-harvesting systems.. Mol Biol Evol.

[B6] Urbach E, Robertson DL, Chisholm SW (1992). Multiple evolutionary origins of prochlorophytes within the cyanobacterial radiation.. Nature.

[B7] Partensky F, Hess WR, Vaulot D (1999). *Prochlorococcus*, a marine photosynthetic prokaryote of global significance.. Microbiol Mol Biol Rev.

[B8] Moore LR, Rocap G, Chisholm SW (1998). Physiology and molecular phylogeny of coexisting *Prochlorococcus *ecotypes.. Nature.

[B9] Moore LR, Chisholm SW (1999). Photophysiology of the marine cyanobacterium *Prochlorococcus*: Ecotypic differences among cultured isolates.. Limnol Oceanogr.

[B10] Dufresne A, Salanoubat M, Partensky F, Artiguenave F, Axmann I, Barbe V, Duprat S, Galperin M, Koonin EV, Le Gall F (2003). Genome sequence of the cyanobacterium *Prochlorococcus marinus *SS120, a nearly minimal oxyphototrophic genome.. Proc Natl Acad Sci USA.

[B11] Palenik B, Brahamsha B, Larimer FW, Land M, Hauser L, Chain P, Lamerdin J, Regala R, Allen RE, McCarren J (2003). The genome of a motile marine *Synechococcus*.. Nature.

[B12] Rocap G, Larimer FW, Lamerdin J, Malfatti S, Chain P, Ahlgren NA, Arellano A, Coleman M, Hauser L, Hess WR (2003). Genome divergence in two *Prochlorococcus *ecotypes reflects oceanic niche differentiation.. Nature.

[B13] Dufresne A, Garczarek L, Partensky F (2005). Accelerated evolution associated with genome reduction in a free-living prokaryote.. Genome Biol.

[B14] Ohmori M, Ikeuchi M, Sato N, Wolk P, Kaneko T, Ogawa T, Kanehisa M, Goto S, Kawashima S, Okamoto S (2001). Characterization of genes encoding multi-domain proteins in the genome of the filamentous nitrogen-fixing Cyanobacterium *Anabaena *sp. strain PCC 7120.. DNA Res.

[B15] Mizuno T, Kaneko T, Tabata S (1996). Compilation of all genes encoding bacterial two-component signal transducers in the genome of the cyanobacterium, *Synechocystis *sp. strain PCC 6803.. DNA Res.

[B16] Gottesman S (2004). The small RNA regulators of *Escherichia coli*: roles and mechanisms.. Annu Rev Microbiol.

[B17] Wagner EG, Simons RW (1994). Antisense RNA control in bacteria, phages, and plasmids.. Annu Rev Microbiol.

[B18] Lankenau S, Corces VG, Lankenau DH (1994). The *Drosophila micropia *retrotransposon encodes a testis-specific antisense RNA complementary to reverse transcriptase.. Mol Cell Biol.

[B19] Morfeldt E, Taylor D, von Gabain A, Arvidson S (1995). Activation of alpha-toxin translation in *Staphylococcus aureus *by the trans-encoded antisense RNA, RNAIII.. EMBO J.

[B20] Lenz DH, Mok KC, Lilley BN, Kulkarni RV, Wingreen NS, Bassler BL (2004). The small RNA chaperone Hfq and multiple small RNAs control quorum sensing in *Vibrio harveyi *and *Vibrio cholerae*.. Cell.

[B21] Sledjeski DD, Gupta A, Gottesman S (1996). The small RNA, DsrA, is essential for the low temperature expression of RpoS during exponential growth in *Escherichia coli*.. EMBO J.

[B22] Altuvia S, Weinstein-Fischer D, Zhang A, Postow L, Storz G (1997). A small, stable RNA induced by oxidative stress: role as a pleiotropic regulator and antimutator.. Cell.

[B23] Wassarman KM, Repoila F, Rosenow C, Storz G, Gottesman S (2001). Identification of novel small RNAs using comparative genomics and microarrays.. Genes Dev.

[B24] Argaman L, Hershberg R, Vogel J, Bejerano G, Wagner EG, Margalit H, Altuvia S (2001). Novel small RNA-encoding genes in the intergenic regions of *Escherichia coli*.. Curr Biol.

[B25] Rivas E, Klein RJ, Jones TA, Eddy SR (2001). Computational identification of noncoding RNAs in *E. coli *by comparative genomics.. Curr Biol.

[B26] Eddy SR (2001). Non-coding RNA genes and the modern RNA world.. Nat Rev Genet.

[B27] Vogel J, Bartels V, Tang TH, Churakov G, Slagter-Jäger JG, Hüttenhofer A, Wagner EGH (2003). RNomics in *Escherichia coli *detects new sRNA species and indicates parallel transcriptional output in bacteria.. Nucleic Acids Res.

[B28] Zhang A, Wassarman KM, Rosenow C, Tjaden BC, Storz G, Gottesman S (2003). Global analysis of small RNA and mRNA targets of Hfq.. Mol Microbiol.

[B29] Valentin-Hansen P, Eriksen M, Udesen C (2004). The bacterial Sm-like protein Hfq: a key player in RNA transactions.. Mol Microbiol.

[B30] Gaudin C, Zhou X, Williams KP, Felden B (2002). Two-piece tmRNA in cyanobacteria and its structural analysis.. Nucleic Acids Res.

[B31] Keiler KC, Shapiro L, Williams KP (2000). tmRNAs that encode proteolysis-inducing tags are found in all known bacterial genomes: A two-piece tmRNA functions in *Caulobacter*.. Proc Natl Acad Sci USA.

[B32] Washietl S, Hofacker IL, Stadler PF (2005). Fast and reliable prediction of noncoding RNAs.. Proc Natl Acad Sci USA.

[B33] Washietl S, Hofacker IL (2004). Consensus folding of aligned sequences as a new measure for the detection of functional RNAs by comparative genomics.. J Mol Biol.

[B34] Complete Results for Non-coding RNA Screening of Cyanobacteria. http://itb.biologie.hu-berlin.de/~kensche/ncRNA_05/index.htm.

[B35] Zengel JM, Lindahl L (1994). Diverse mechanisms for regulating ribosomal protein synthesis in *Escherichia coli*.. Prog Nucleic Acid Res Mol Biol.

[B36] Lindahl L, Zengel JM (1986). Ribosomal genes in *Escherichia coli*.. Annu Rev Genet.

[B37] Barry G, Squires C, Squires CL (1980). Attenuation and processing of RNA from the *rplJL-rpoBC *transcription unit of *Escherichia coli*.. Proc Natl Acad Sci USA.

[B38] Winkler W, Nahvi A, Breaker RR (2002). Thiamine derivatives bind messenger RNAs directly to regulate bacterial gene expression.. Nature.

[B39] Nahvi A, Barrick JE, Breaker RR (2004). Coenzyme B12 riboswitches are widespread genetic control elements in prokaryotes.. Nucleic Acids Res.

[B40] Tjaden B, Saxena RM, Stolyar S, Haynor DR, Kolker E, Rosenow C (2002). Transcriptome analysis of *Escherichia coli *using high-density oligonucleotide probe arrays.. Nucleic Acids Res.

[B41] Reyes JC, Muro-Pastor MI, Florencio FJ (1997). Transcription of glutamine synthetase genes (*glnA *and *glnN*) from the cyanobacterium *Synechocystis *sp. strain PCC 6803 is differently regulated in response to nitrogen availability.. J Bacteriol.

[B42] Golden SS, Brusslan J, Haselkorn R (1986). Expression of a family of *psbA *genes encoding a photosystem II polypeptide in the cyanobacterium *Anacystis nidulans *R2.. EMBO J.

[B43] Tegmark K, Morfeldt E, Arvidson S (1998). Regulation of agr-dependent virulence genes in *Staphylococcus aureus *by RNAIII from coagulase-negative staphylococci.. J Bacteriol.

[B44] Zago MA, Dennis PP, Omer AD (2005). The expanding world of small RNAs in the hyperthermophilic archaeon *Sulfolobus solfataricus*.. Mol Microbiol.

[B45] Fuller NJ, Marie D, Partensky F, Vaulot D, Post AF, Scanlan DJ (2003). Clade-specific 16S ribosomal DNA oligonucleotides reveal the predominance of a single marine *Synechococcus *clade throughout a stratified water column in the Red Sea.. Appl Environ Microbiol.

[B46] Watanabe T, Sugiura M, Sugita M (1997). A novel small stable RNA, 6Sa RNA, from the cyanobacterium *Synechococcus *sp. strain PCC6301.. FEBS Lett.

[B47] Barrick JE, Sudarsan N, Weinberg Z, Ruzzo WL, Breaker RR (2005). 6S RNA is a widespread regulator of eubacterial RNA polymerase that resembles an open promoter.. RNA.

[B48] Damerval T, Houmard J, Guglielmi G, Csiszar K, Tandeau de Marsac N (1987). A developmentally regulated *gvpABC *operon is involved in the formation of gas vesicles in the cyanobacterium *Calothrix *7601.. Gene.

[B49] Synechococcus sp. Genome Analysis. http://www.genoscope.cns.fr/externe/English/Projets/Projet_HP/organisme_HP.html.

[B50] Trotochaud AE, Wassarman KM (2004). 6S RNA function enhances long-term cell survival.. J Bacteriol.

[B51] Vogel J, Axmann IM, Herzel H, Hess WR (2003). Experimental and computational analysis of transcriptional start sites in the cyanobacterium *Prochlorococcus *MED4.. Nucleic Acids Res.

[B52] Chisholm lab protocols. http://web.mit.edu/chisholm/www/.

[B53] Garcia-Fernandez JM, Hess WR, Houmard J, Partensky F (1998). Expression of the *psbA *gene in the marine oxyphotobacteria *Prochlorococcus *spp.. Arch Biochem Biophys.

[B54] Logemann J, Schell J, Willmitzer L (1987). Improved method for the isolation of RNA from plant tissues.. Anal Biochem.

[B55] Sambrook J, Fritsch EF, Maniatis T (1989). Molecular Cloning A Laboratory Manual.

[B56] Altschul SF, Gish W, Miller W, Myers EW, Lipman DJ (1990). Basic local alignment search tool.. J Mol Biol.

[B57] The DDBJ/EMBL/GenBank Feature Table: Definition. http://www.ncbi.nlm.nih.gov/collab/FT/.

[B58] Thompson JD, Higgins DG, Gibson TS (1994). ClustalW: improving the sensitivity of progressive multiple sequence alignment through sequence weighting, position-specific gap penalties and weight matrix choice.. Nucleic Acids Res.

[B59] Zuker M (2003). Mfold web server for nucleic acid folding and hybridization prediction.. Nucleic Acids Res.

[B60] Luck R, Graf S, Steger G (1999). ConStruct: a tool for thermodynamic controlled prediction of conserved secondary structure.. Nucleic Acids Res.

[B61] Sanangelantoni AM, Tiboni O (1993). The chromosomal location of genes for elongation factor Tu and ribosomal protein S10 in the cyanobacterium *Spirulina platensis *provides clues to the ancestral organization of the str and S10 operons in prokaryotes.. J Gen Microbiol.

[B62] Zengel JM, Mueckl D, Lindahl L (1980). Protein L4 of the *E. coli *ribosome regulates an eleven gene r protein operon.. Cell.

[B63] Li SC, Squires CL, Squires C (1984). Antitermination of *E. coli *rRNA transcription is caused by a control region segment containing lambda *nut*-like sequences.. Cell.

[B64] Condon C, Squires C, Squires CL (1995). Control of rRNA transcription in *Escherichia coli*.. Microbiol Rev.

[B65] Christensen T, Johnsen M, Fiil NP, Friesen JD (1984). RNA secondary structure and translation inhibition: analysis of mutants in the *rplJ *leader.. EMBO J.

[B66] Vitreschak AG, Rodionov DA, Mironov AA, Gelfand MS (2003). Regulation of the vitamin B12 metabolism and transport in bacteria by a conserved RNA structural element.. RNA.

[B67] Ermolaeva MD, Khalak HG, White O, Smith HO, Salzberg SL (2000). Prediction of transcription terminators in bacterial genomes.. J Mol Biol.

[B68] Steward KL, Linn T (1992). Transcription frequency modulates the efficiency of an attenuator preceding the *rpoBC *RNA polymerase genes of *Escherichia coli*: possible autogenous control.. Nucleic Acids Res.

[B69] Branlant C, Krol A, Machatt A, Ebel JP (1981). The secondary structure of the protein L1 binding region of ribosomal 23S RNA. Homologies with putative secondary structures of the L11 mRNA and of a region of mitochondrial 16S rRNA.. Nucleic Acids Res.

[B70] Huynen MA, Bork P (1998). Measuring genome evolution.. Proc Natl Acad Sci USA.

