# Supplementary material for: Identification of cyanobacterial non-coding RNAs by comparative genome analysis
Source: Genome Biol. 2005 Aug 17;6(9):R73. doi: 10.1186/gb-2005-6-9-r73 (PMC1242208; doi:10.1186/gb-2005-6-9-r73)
Supplement: Additional data file 1 — Figures showing the determination of half-lifes for several ncRNAs (Figure S9) and the composition of cluster 53 (Figure S10). [file gb-2005-6-9-r73-S1.pdf]

Supplemental Figures

Suppl. Fig. S9: Determination of half lifes for Yfr1, Yfr2 and Yfr5 through Yfr7 in *Prochlorococcus* MED4. The time after the addition of rifampicin in minutes is given on top. Hybridization to 5S rRNA served as control.

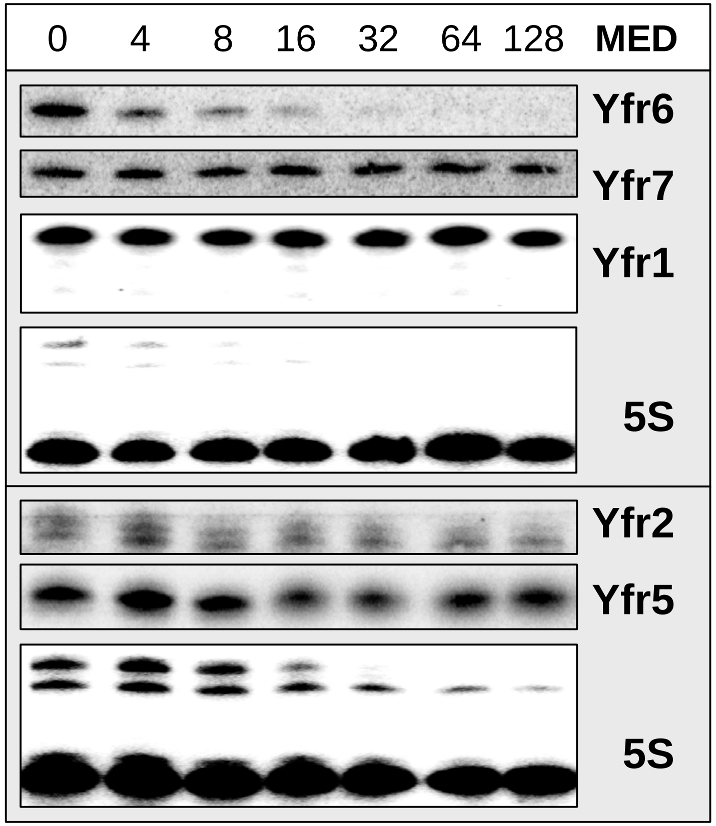

Suppl. Fig. S10: Composition of cluster 53. It contains nine sequence: 2 from MED4, 2 from SS120, 1 from MIT9313 and 4 from WH8102. Among them are Yfr6 from MED4 and SS120. The seven other sequences each have only about 40 nucleotide positions in common with yfr6 **(A)**. These sequences included mRNAs for two previously not annotated genes in MED4 and MIT9313 (PMM3822n and PMT3904n), the 5' UTR sequences for the genes Pro0415 (in SS120), SYNW1950 and SYNW2450 (in WH 8102) as well as two more sequences in WH 8102, (27_W1i1019 and 6_W1i283), which were not further characterized. A high sequence similarity exists only in the regions 5’ to annotated or not annotated reading frames for short peptides (possible start codons are red). Shown is the region from a multiple sequence alignment with the top similarity. Single guanosin nucleotides experimentally determined as initiation sites of transcription for Yfr6 and PMM3822n are shown in red, too.
Despite the localization of DNA sequence similarity in possible 5’ UTR it is possible to align the derived amino acid sequences of the downstream encoded hypothetical proteins **(B)**.

A

```
                         *        20         *        40         *        60         *        80
Pro0415    : ATTTTGTAGCGATTGCTACAAAAACGTTCACCCTGAT-A--CT---TCAGGGCGCAGACCCAATAAGGCCACGGAACGGGGAC :  77
PMT3904n   : GATTTGTAGCGATAGCTACCAAAACGTTCACCCTGATCA--TT---GCAGGGCGCAGTTCGAGCCAAGCCATGGAACGGGGAC :  78
SYNW1950   : ATTCGGTAGCAGATGCTACTAAT-CGTTCACTCCGTT-A--TC---GCGGAGCGCAG-TCAAGCCAGGCCATGGAACGGGGAC :  75
SYNW2450   : ATTTTGCAGCAACTGCTACAG-A-CGTTCACCTCGCC-AGCCTCG-GCAAGGCGCAGTTCGACTCAGGCCATGGAACGGGGAC :  79
27 W1i1019 : GATGTGTAGCACCTGCTACTGCA-CGTTCACCTCACC-A--CT-G-GCGAGGCGCAGTTCGACTCAGGCCATGGAACGGGGAC :  77
MED4 yfr6  : AATTTGTAGCAACCGCTACCAAAACGTTCAACTTG-T-A--ATAATGCAAGCCGCAAATCGACTTAAGCCATGGAACGGGGAC :  79
SS120 yfr6 : ATTTTGTAGCAACAGCTACTAAAACGTTCAACTCT-T-A--TTAG----AGCCGCAGTTCGACTCAAGCCATGGAACGGGGAC :  75
PMM3822n   : AAAAAGTAGCTATTGCTAC-ATTTCGTTCACCTTCTTTAG--------AAGGCGCAGTTCGAGTCATACCAGGGAACGGGGGA :  74
6_W1i283   : ATTATGTAGCTAGCGCTACCAAG-CGTTCACCTCACT----TT---TCGAGGCGCAGCTCGATCCAAGCCATGGAACGGGGGC :  75

                    *        100         *        120         *
Pro0415    : CTTACAAAT-TAGA--GGA-CACTGCAA-ATGACAAACCTTGTTTAT : 119
PMT3904n   : -TTGGACGT-CATTTCGGA-CTCTGAATCATGACCACGCTTCTTTAT : 122
SYNW1950   : -CTGGATTT-CTTCGAGGAACACC-CA--ATGACAACCCTTCTGTAT : 117
SYNW2450   : -CTGAGCTTGCTTCGAGGA--ACC--ACCATGAC------GACTCTT : 115
27 W1i1019 : -CTGAGCTTGCTTCGAGGA--ACC-CATCATGAC---CCTGACCTAT : 117
MED4 yfr6  : -TTGGGCGA-AACCGGAGA-AAAT--AG-ATGACA---CTTATCTAC : 117
SS120_yfr6 : -TTGAGCTA-ACCCGGAGA-CAAC--A--ATGTCA---CTAACCTAC : 112
PMM3822n   : --TGGCCGT--TTTG-TCA-CATCGAAACATGACTACTTTAACTTAC : 115
6_W1i283   : -TTGGGCTC--TGC-AGGA--GCTGTGTGATGACTTCTTTGTATTGG : 116
```

B

```
                       *        20         *        40         *        60
SYNW1950   : MTTLLYRGNEYVQQNK-AAETSPVQLTYRRNVYQGRLNEAALHNVALTGVQLTYRGVSYTR-------- : 60
SYNW2450   : MTTLLYRGHAYQQVKD-AAQQQGVQLTYRRNVYQARQ-----ADVRQAQVQLTYRGVSYLR-------- : 55
PMM3822n   : MTTLTYRGKNYVQNKK-AAKKQPVELTYRRNVYTNRMDD---ASSSNEKAELNYRGAKYTK-------- : 57
PMT3904n   : MTTLLYRGQQYAQHKE-VAPKQLVELTYRRTVYANNK-----LKAAQTHPVLTYRGQEYQK-------- : 55
PRO0415    : MTNLVYRGSNYVQHKE-AAKKQYVELTYRRNVYSNRK-----KKASTDHAQLTYRGIPYQI-------- : 55
6 W1i283   : MTSLYWNGLAFAPVNKTAAKKEAVILRLETALMAPDA-----------TPSLKYRGISYLRQNGFVATL : 58
W1i1019    : MT-LTYRGQKYVQNNA-AATNS--------------K-----------RPVLVYRGHKVAQ-------- : 34
SS120 yfr6 : MS-LTYRGQKYTQHKA-VVEK---------------K-----------HVLLTYRGKRYSI-------- : 33
MED4__yfr6 : MT-LIYRGQKYVQNKV-AAKKQ--------------------------HTELTYRGKAYTS-------- : 33
```
