# Supplementary material for: Identification of cyanobacterial non-coding RNAs by comparative genome analysis
Source: Genome Biol. 2005 Aug 17;6(9):R73. doi: 10.1186/gb-2005-6-9-r73 (PMC1242208; doi:10.1186/gb-2005-6-9-r73)
Supplement: Additional data file 2 — Tables listing the genome versions used in this study and details of intergenic regions (Table S4), the parameters for the initial local alignment of intergenic spacer regions, the clustering step and ALIFOLDZ (Table S5) and the sequences of oligonucleotides used in this study (Table S3). [file gb-2005-6-9-r73-S2.pdf]

Table S3. Genome versions used in this study and details of intergenic regions. Sequences were downloaded from NCBI (ftp://ftp.ncbi.nih.gov/genbank/genomes/Bacteria).

Strain	NCBI acc. #	Version	Length	No of Genes	%GC	No of IGRs >49 nt	IGR total nt (% of genome)	IGR %GC
MED4	NC_005072.1	19-AUG-2003	1,657,990	1,716	30.8	793	192,524 (10.8%)	24.8
MIT 9313	NC_005071.1	21-AUG-2003	2,410,873	2,275	50.7	1,308	427,459 (17.1%)	44.1
SS120	NC_005042.1	14-AUG-2003	1,751,080	1,882	36.4	989	192,250 (10.0%)	32.2
WH 8102	NC_005070.1	19-AUG-2003	2,434,428	2,528	59.4	1,001	234,454 (8.7%)	52.0

Table S4. Parameters for the initial local alignment of intergenic spacer regions, the clustering step and AlifoldZ.

**BLASTN**	**parameter value**
gap open	2
gap extension	2
match	2
mismatch	-2
e-value cutoff	1,00E-05
**Import of HSPs**	
e-value cutoff	1,00E-10
**Unify overlapping hits**	
max. reciprocal coverage	85%
**BLASTN clustering**	
gap open	2
gap extension	10
match	2
mismatch	-2
query strand ('S' option)	1 (=top)
e-value cutoff	1,00E-10
**Unify of overlapping hits / cluster**	
max. reciprocal coverage	10%
**CLUSTALW alignment**	default
**ALIFOLDZ / RNAfold**	
temperature	24°C
background size (shufflings)	100

Suppl. Table S5. Sequences of oligonucleotides used in this study.

	Name	Sequence (5‘ → 3‘)	Experiment
**control**	PMM3822n	TCC CTG GTA TGA CTC GAA CTG C	hybridisation mRNA of PMM3822n Med4
**known and annotated RNA genes**			
	5S	GGC ATT GAG CTA TTT TCT CAG GGG GCT	hybridisation 5S rRNA
	t_ser	CGG AGA GGG AGG GAT TCG AAC	hybridisation tRNA-Ser
	tm5	CGG AAT CGA ACC GCT GTC CGA	hybridisation tmRNA (5’end):
	tm3MITWH	TGA CGG GAG AAA CGA ACG ATG TTG T	hybridisation tmRNA (3’end) in MIT9313 and WH8102
	tm3MS	GCT GTT TGA CGG GAG AAA CTA ACG AT	hybridisation tmRNA (3’end) in MED4 and SS120
	sc	CCT TCC GRT CCT GAC CAG RTT T	hybridisation scRNA
**predicted and verified RNA genes**			
	y1M	CGA TTT GGT GTG TGA GGA GTA TGG	hybridisation Yfr1 in MED4
	y1aM	ACA GAC TTA AAA AAG CCC GAT AA	5'RACE reverse transcription Yfr1 MED4
	y1nestM	GCG ATT TGG TGT GTG AGG AGT ATG GGG	5'RACE PCR Yfr1 MED4
	y1MIT	CGG TGT GTG TGA GGA GTG TGT G	hybridization Yfr1 in MIT9313

	y1WH	GGG GTG TGA GGA GTG TGT GG	hybridization Yfr1 in WH8102
	y_genM	CCA CTG TTT CAG TAA AAC CTC TCC TAC	Yfr2-Yfr5 consensus primer for hybridization in MED4
	y2aM	ATA GAG GTC TTT CCT TGG TTT AA	5'RACE reverse transcription, hybridisation Yfr2 MED4
	y3aM	ATT TCT AGA GAT CTT TAT ATA GTT TTA CT	5'RACE reverse transcription, hybridisation Yfr3 MED4
	y4aM	TCT AGA GGT CTT TTT CTG GTT TT	5'RACE reverse transcription, hybridisation Yfr4 MED4
	y5aM	AAG GGG CCT AAA TTG GTT CTA	5'RACE reverse transcription, hybridisation Yfr5 MED4
	y2nestM	CTA AAT CAA GTG TTT CCT TGT TTC CAC TGT TTC AG	5'RACE PCR Yfr2 MED4
	y3nestM	AAC CAA GTG TTT CCT TGT TTC CAC TGT TTC AGT AAA A	5'RACE PCR Yfr3 MED4
	y4nestM	CCG AAT CAA GTG TTT CCT TGT TTC CAC TGT TTC	5'RACE PCR Yfr4 MED4
	y5nestM	CGA GTG TTT CCT TGT TTC CAC TGA TTT AAT AAA ACC	5'RACE PCR Yfr5 MED4
	y2to5M	GTG TTT CCT TGT TTC CAC TG	Yfr2-Yfr5 consensus primer for hybridization in MED4
	y2S	GTG TTT CCT AGT TTC CAC TTT T	hybridization Yfr2 in SS120
	y2MIT	GTG TTT CCT TGT TTC CAC TC	hybridization Yfr2 in MIT9313
	y6MS	GTT GAA CGT TTT RGT AGC KGT TGC TAC	hybridisation of Yfr 6 in MED4 and SS120
	y6aM	CTG TAT GCT GTT TTT TTG CAG C	5'RACE Yfr6 reverse transcription in MED4

	y6aS	CAT GCT TCT TTT CAA CTA CTG C	5'RACE Yfr6 reverse transcription in SS120
	y6nestM	GTT TTG GAC GTA TTT TTG TCC TCT GTA GAT AAG TGT CAT C	5'RACE PCR Yfr6 in MED4
	y6nestS	GTT GAG TGT ACT TTT GAC CTC TGT AGG TTA GTG AC	5'RACE PCR Yfr6 in SS120
	y7aMS	AAG CCA GAA GAG GAG TCA A	5'RACE Yfr7 reverse transcription MED4 and SS120
	y7nest1M	GCT TTC GTT TCC AAT TAC AGG ATT GGT TTA CGT CTA CAT	5'RACE PCR Yfr7 MED4
	y7nest2M	CAC ATA AAG AAG AAC TTG ACC AAA GAT CCA GTA TGT AAA TA	5'RACE PCR Yfr7 MED4
	y7nest1S	TGA TCC ATT TCC AGT TGC ATG ACT GGT TTA CTT CAC	5'RACE PCR Yfr7 SS120
	y7nest2S	CTT AAA AGG TCA TCA CCA ACA AAA CAG TTC TCT TGC ATA	5'RACE PCR Yfr7 SS120
	y7MS	CCT CAA GTC GAA AAA GAG TCA GAT CAG A	hybridisation Yfr7 MED4 and SS120
	y7MS_2	GTC GAA AAA GAG TCA GAT CAG AGC AC	hybridisation Yfr7 Med4 and SS120
	y7MIT9312	GTC GAA AGA AAG TCA GAT CAG AGC AC	hybridisation Yfr7 MIT 9312
	y7MITWH	TCG AAA GAG AGT CAG ATC AGA GCA C	hybridisation Yfr7 MIT9313, WH8102 and WH 7803
**not yielding signals**			
	y1S	TTC TGC AGC AAT CTA AAT TTT TAA GAG AAG AAA AAT AA	hybridization *guaB-trxA* intergenic region for the presence of Yfr1 in SS120
	ID273MSt44rev	CCC GAA ACA GTC AGA TGT G	hybridisation to putative t44 RNA in MED4 and SS120

	ID273WHt44rev	CCC GAA ACG GGC AGG	hybridisation to putative t44 RNA in WH8102
	ID273MITt44rev	CCC GAA ACG GAC AGG T	hybridisation to putative t44 RNA in MIT9313
	ID217Mrev	CAG GGG TTG ATG GGT C	hybridisation to candidate RNA CLID217 in MED4
	ID217Mfw	GAC CCA TCA ACC CCT G	hybridisation to candidate RNA CLID217 in MED4
	ID102Mfw	CCA CAG AAA CTA CTA CTA CTA TAT AAA TTC ATT ATT AG	hybridisation to candidate RNA CLID102 in MED4
	ID102Mrev	CTA ATA ATG AAT TTA TAT AGT AGT AGT AGT TTC TGT GG	hybridisation to candidate RNA CLID102 in MED4
	ID185Mfw	AGA TCG TAT TCA TCC ATG GTT AAG TAA AGT TAA A	hybridisation to candidate RNA CLID185 in MED4
	ID185Mrev	TTT AAC TTT ACT TAA CCA TGG ATG AAT ACG ATC T	hybridisation to candidate RNA CLID185 in MED4
	ID66Mfw	TAT GAG TGA ATA TGA TAA TGA ATA TCA ATA GCA ATA TCA A	hybridisation to candidate RNA CLID66 in MED4
	ID66Mrev	TTG ATA TTG CTA TTG ATA TTC ATT ATC ATA TTC ACT CAT A	hybridisation to candidate RNA CLID66 in MED4
	ID194Mfw	GTA GGA GAG GTT TTA CTG AAA CAG TGG	hybridisation to candidate RNA CLID194 in MED4
	ID257fw	CCCCATACTCCTCACACAC	hybridisation to candidate RNA CLID257 in MED4
	ID53Sfw	GTA GCA ACA GCT ACT AAA ACG TTC AAC	hybridisation to candidate RNA CLID53 in SS120
	ID53Mfw	GCA ACC GCT ACC AAA ACG T	hybridisation to candidate RNA CLID53 in MED4
	ID51Sfw	GTC ATG CAA CTG GAA ATG GAT CAT TAC	hybridisation to candidate RNA CLID51in SS120

	ID51Mfw	GTA ATT GGA AAC GAA AGC CCA	hybridisation to candidate RNA CLID51in MED4
